# Supplementary material for: Structural and functional insights into the bona fide catalytic state of Streptococcus pyogenes Cas9 HNH nuclease domain
Source: eLife. 2019 Jul 30;8:e46500. doi: 10.7554/eLife.46500 (PMC6706240; doi:10.7554/eLife.46500)
Supplement: Supplementary file 1. [file elife-46500-supp1.docx]

**Table S1 | Summary of the free energies of the pseudo-active and active Cas9 complex systems with (+) and without (-) Mg^2+^ bound at the HNH domain catalytic interface estimated by the end-point MM-GBSA approach (kcal/mol)**

|  | **+ Mg^2+^** | **- Mg^2+^** | **ΔΔG** |
| --- | --- | --- | --- |
| **ΔG(pseudo-active)** | -64538.9 (6.7) | -63947.6 (6.7) | -591.3 |
| **ΔG(active)** | -64513.5 (4.8) | -63954.8 (4.8) | -558.7 |
| **ΔΔG** | -25.4 |  |  |

Values are Mean (SEM) calculated from a sample of 2,000 structures.
