# Supplementary material for: Structural and functional insights into the bona fide catalytic state of Streptococcus pyogenes Cas9 HNH nuclease domain
Source: eLife. 2019 Jul 30;8:e46500. doi: 10.7554/eLife.46500 (PMC6706240; doi:10.7554/eLife.46500)
Supplement: Supplementary file 2. [file elife-46500-supp2.docx]

**Table S2 | List of primers used to construct SpyCas9^D861A^ and SpyCas9^N863A^**

| **Construct** | **Mutagenic primers** | **Method** |
| --- | --- | --- |
| SpyCas9^D861A^ | 5′-ACGCGTTCTGCAAAAAATCGTGGTAAATCGGATAACGTTCCA-3′  5′-ACGATTTTTTGCAGAACGCGTTAAGACCTTATTGTCTATTGA-3′ | SLIC (Ref. (***Scholz et al., 2013***) |
| SpyCas9^N863A^ | 5′-TCTGATAAAGCGCGTGGTAAATCGGATAACGTTCCAAGTGAA-3′  5′-TTTACCACGCGCTTTATCAGAACGCGTTAAGACCTTATTGTC-3′ | SLIC (Ref. (***Scholz et al., 2013***) |
| Non-target strand  (NT) | 5′-ATCCTGCGCTGGTTGATTTCTTCTTGCGCTTTTTGGGGAATTCA  CTGGCCGTCG -3′ | N/A |
| Target strand (T) | 5’-CGACGGCCAGTGAATTCCCCAAAAAGCGCAAGAAGAAATCAA  CCAGCGCAGGAT -3′ | N/A |

Ref.: Scholz J, Besir H, Strasser C, and Suppmann S. 2013. A new method to customize protein expression vectors for fast, efficient and background free parallel cloning. *BMC Biotechnol* **13**: 12.
